# The unique role of meaning in life in the relationships between trait awe, subjective well‐being, and prosocial tendency: A network analysis

**DOI:** 10.1002/pchj.733

**Published:** 2024-02-16

**Authors:** Yichao Lv, Qian Xu, Qihui Tang, Yanqiang Tao, Chao Zhang, Xiangping Liu

**Affiliations:** ^1^ Faculty of Psychology Beijing Normal University Beijing China; ^2^ Beijing Key Laboratory of Applied Experimental Psychology National Demonstration Center for Experimental Psychology Education Beijing China; ^3^ School of Psychology Nanjing Normal University Nanjing China; ^4^ School of Education Science Shanxi Normal University Taiyuan China

**Keywords:** meaning in life, network analysis, prosocial tendency, subjective well‐being, trait awe

## Abstract

Although individuals with higher trait awe (the tendency to experience awe) are known to be happier and more prosocial, there is limited understanding of the mechanisms underlying these complex relationships. This study uses network analysis to explore dimension‐level relationships between trait awe, meaning in life, subjective well‐being (SWB), and prosocial tendency in a joint network and to explore the bridging role of meaning in life in the network. A total of 538 adults (53.2% females; *M*_age_ = 19.86 ± 1.51) completed the survey. The network revealed unique and intricate connections between the dimensions of trait awe, meaning in life (i.e., the presence of and the search for meaning, abbreviated as POM and SFM), subjective happiness and life satisfaction (SWB), and prosocial tendency (i.e., willingness to donate money and volunteer time). Trait awe exhibited direct links to subjective happiness, life satisfaction, and prosocial tendency to donate money. Moreover, through POM and SFM, trait awe also exhibited indirect links to each dimension of SWB and prosocial tendency. Within the global network, POM was further identified as acting as a bridge node with the highest bridge strength and closeness, indicating that POM could efficiently transmit influences within the entire network. These findings highlight the distinct contributions of meaning in life to understanding the relationships between trait awe, SWB, and prosocial tendency, and provide valuable insights for improving SWB and fostering prosocial tendencies.

## INTRODUCTION

Awe is a complex emotion that arises when individuals confront physically or conceptually vast stimuli that transcend their existing cognitive structures (Keltner & Haidt, 2003). Keltner and Haidt (2003) proposed two core appraisals of awe: perceived vastness and the need for accommodation. Perceived vastness refers to the size or magnitude of stimuli in terms of physical size, ability, power, virtue, or complexity. The need for accommodation refers to the need to adjust or even reconstruct one's current cognitive structures. In daily life, awe can be inspired by interpersonal elements such as virtue, virtuosity, and excellent character, as well as by extraordinary natural phenomena such as mountains, storms, and the universe (Bai et al., 2017; Lv et al., 2023). Despite being mixed with negative emotions such as fear and anxiety, awe is generally considered a distinct positive emotion (Gordon et al., 2017).

Trait awe, also known as dispositional awe, refers to an individual's disposition or tendency to experience awe, particularly positive awe. Unlike transient state awe, which can be manipulated, trait awe is relatively stable. Individuals higher in trait awe may experience awe more frequently and intensely in everyday life because they are more open to novel stimuli, more sensitive to deep emotions and experiences, and more receptive to new ways of thinking and perspectives (Shiota et al., 2006). Guided by the broaden‐and‐build theory (Fredrickson et al., 2008), awe, as a unique positive emotion, is assumed to broaden one's thought‐action repertoires, further contributing to the development of long‐lasting personal resources (e.g., subjective well‐being, SWB) and increasing one's willingness or behavior to cooperate, share, and help others (i.e., one's prosocial tendency). In addition, numerous studies have demonstrated the self‐transcendent nature of awe, supporting the prototype theory of awe (Keltner & Haidt, 2003). Transiently elicited awe (or state awe) has been found to promote various aspects of SWB and prosocial tendencies (Guan et al., 2019; Piff et al., 2015; Rudd et al., 2012). Following this, researchers have also examined the positive predictions of trait awe on SWB and prosocial tendency, as well as the mediation of meaning in life (Fu et al., 2022; Zhao et al., 2019; Zhao & Zhang, 2022). Owing to the self‐transcendent nature of trait awe, people tend to perceive (i.e., presence of meaning, POM) and pursue (i.e., search for meaning, SFM) meaning in life and thus tend to be happier and more prosocial. Specifically, using a latent variable model, Zhao et al. (2019) found that trait awe could predict more SWB (observable variable: subjective happiness and life satisfaction) through a higher level of meaning in life (observable variable: POM and SFM). Recently, using a longitudinal mediation analysis, Fu et al. (2022) further found that trait awe could predict a higher level of POM, which in turn predicted prosocial behaviors six months later. Moreover, other researchers also suggest that SFM may be another mediator between trait awe and prosocial tendency (Dakin et al., 2021; Rivera et al., 2020).

However, these studies have some limitations and so necessitate further consideration and improvement. First, the relationships at the dimension level between trait awe, meaning in life, SWB, and prosocial tendency remain unclear. Prior research often focused on relationships between variables or latent variables, neglecting the relationships between sub‐dimensions or observable indicators. Using a latent variable to reflect observable indicators (Zhao et al., 2019) or aggregating scores of sub‐dimensions as a variable score (Fu et al., 2022) has, to some extent, blurred the distinctions between dimensions or observable indicators. Taking meaning in life as an example, the two dimensions—POM and SFM—show fundamental differences and distinct relationships with other variables (Chan, 2016; Liu & Gan, 2010; Steger et al., 2008). POM, in particular, exhibits stronger associations with higher levels of SWB and prosocial behaviors compared with SFM (Li et al., 2021; Xie et al., 2023). These notable differences underscore the necessity to explore relationships between these variables at the dimension level. Specifically, we should delve into the specific relationships between trait awe and POM and SFM, as well as into the respective mediating effects of POM and SFM between trait awe and SWB and prosocial tendency. On the other hand, prior models only examined how trait awe separately predicts SWB or prosocial tendencies, thereby overlooking the potential interaction between the two predicted variables. Indeed, there are positive feedback loops between SWB and prosocial tendencies (Hui, 2022; Xiong et al., 2023), which are mutually reinforcing. Integrating these variables into a model provides a more comprehensive view of the emotional, cognitive, and attitudinal benefits of awe, while also increasing the model's face validity. To address these issues, we introduce network analysis in this study to elucidate the complex relationships between variables and examine the roles of key variables, thereby partially compensating for the limitations of previous research (Fried & Cramer, 2017).

## NETWORK ANALYSIS

Network analysis is an emerging novel approach to visualizing complex psychological phenomena that arise from the mutual connections between the observable indicators that define them (e.g., items or dimensions; Epskamp et al., 2018). Network analysis suggests that observable indicators are not simple reflections or causes of latent variables but rather are interconnected and mutually reinforce each other. For instance, POM and SFM are not direct assessments of meaning in life but are integral components of it. More importantly, the network analysis approach has the advantage of using graphs to visualize the complex associations among psychological variables. Within the network, communities represent the psychological variables (e.g., meaning in life), nodes represent the items or dimensions of psychological variables (e.g., POM and SFM), and edges represent the correlation or partial correlation between these variant items or dimensions. By comparing the particular edge weights, we could find out whether the node (e.g., the POM) is more connected with a specific node (e.g., subjective happiness) than another node (e.g., the SFM). Notably, the network based on cross‐sectional data does not emphasize the direction of the edges and is therefore suitable for exploring the relationships between certain interacting variables (e.g., SWB and prosocial tendency).

For a network consisting of variant communities or variables, network analysis provides bridge centrality indices, such as bridge strength/expected influence, bridge betweenness, and bridge closeness, to identify the most critical bridge nodes in the network. Similar to in mediation analysis, bridge centrality estimations could help us understand the underlying mechanisms about how the influence of one variable is transmitted through the bridges to another (Jones et al., 2021). By comparing bridge centrality, we can compare the roles of any two nodes in the network. Bridge strength/expected influence quantifies how much influence the bridge nodes transmit between different variables (or communities). Bridge betweenness is used to infer which nodes might frequently act as bridges or mediators in network transactions. Bridge closeness quantifies which nodes can provide the shortest path for the transmission of impacts. Notably, bridge betweenness and bridge closeness may have limited stability in certain psychometric networks, and therefore bridge strength has been most commonly used to identify bridge nodes (Epskamp et al., 2018).

Above all, network analysis can contribute to investigating the specific pathways linking trait awe to the dimensions of meaning in life, SWB, or prosocial tendency, as well as to revealing the underlying mechanisms of how trait awe is related to the development and maintenance of SWB and prosocial tendency. Although there is no direct network evidence supporting the network structure of the above variables and the bridging role of meaning in life, we could still clarify the relationships and potential mechanisms of these variables through previous theoretical and empirical research. In the present study, we use network analysis to explore the complex relationships between trait awe, SWB, and prosocial tendency and to identify the bridging role of meaning in life.

## THE NETWORK OF TRAIT AWE, SWB, AND PROSOCIAL TENDENCY

### Trait awe and SWB

SWB is an important psychological benefit of trait awe. SWB, commonly referred to as “happiness,” encompasses both subjective happiness and life satisfaction, corresponding to an individual's emotional and cognitive evaluations of their overall life, respectively (Diener, 2000; Diener et al., 2003; Lyubomirsky & Lepper, 1999). Subjective happiness involves how happy or unhappy individuals feel about themselves, while life satisfaction involves how satisfied individuals are with their lives overall. In the network, SWB might emerge from the interactions between the two aspects (Blasco‐Belled & Alsinet, 2022). The broaden‐and‐build theory of positive emotions (Fredrickson, 2001) proposes that positive emotions, such as awe, could broaden one's thought‐action repertoires (e.g., attention, cognitive processes, perceptions, and thoughts) and further promote the development of individual psychological resources (e.g., SWB). As a unique positive emotion (Shiota et al., 2006), awe could increase positive affect and life satisfaction more than other general positive emotions (Rudd et al., 2012). Therefore, individuals who experience awe more frequently and intensely and presumably accumulate more awe emotions, may report higher levels of SWB. Some studies have directly supported this assumption (Dong & Ni, 2019; Zhao et al., 2019). For instance, Dong and Ni (2019) found that individuals with higher trait awe tended to experience greater levels of happiness. Zhao et al. (2019) further found that they also tended to exhibit higher levels of life satisfaction. More recently, Zhao and Zhang (2022) found that trait awe could also predict higher levels of psychosocial flourishing, which refers to an optimal and sustained sense of SWB. Therefore, we propose our first hypothesis for this study:

Hypothesis 1 Trait awe is positively connected (or associated) with subjective happiness and life satisfaction in the network model.

### Trait awe and prosocial tendency

Prosocial tendency is another important psychological benefit of trait awe. Prosocial tendency refers to an individual's willingness and preference to engage in behaviors that are generally beneficial to others, such as helping, donating, volunteering, and cooperating (Penner et al., 2005). In empirical studies, donating money and volunteering time are two primary forms of prosocial behavior (Guan et al., 2019; Rudd et al., 2012). However, there is no consensus on whether these two forms are complementary or substitutive, as they may involve different cognitive and decision‐making processes (Costello & Malkoc, 2022; Small & Cryder, 2016). According to the prototype theory of awe (Keltner & Haidt, 2003), awe elicits a sense of a small self that shifts individuals' focus from themselves to the welfare of others and then encourages them to be more generous and prosocial towards others (Chirico & Yaden, 2018). Therefore, individuals with higher trait awe may have higher levels of prosocial tendencies because they may experience more awe. Several studies have examined this positive relationship (Fu et al., 2022; Lin et al., 2020; Piff et al., 2015). Specifically, Piff et al. (2015) found that individual differences in trait awe tended to predict generosity in prosocial giving, even when controlling for other prosocial trait emotions (e.g., love and compassion). Moreover, trait awe has also been found to predict prosocial tendencies in various specific contexts, including public, anonymous, altruistic, compliant, dire, and emotional contexts (Jiao & Luo, 2022; Lin et al., 2020). Therefore, we propose our second hypothesis:

Hypothesis 2 Trait awe is positively connected (or associated) with the willingness to donate money and volunteer time in the network model.

### SWB and prosocial tendency

SWB and prosocial tendency are not only the psychological downstreams of trait awe, but they could also reinforce each other. In other words, individuals might derive more SWB from such prosociality while acting more prosocially through increased SWB (Hui, 2022). Several theories have highlighted such a positive feedback loop. On the one hand, if the prosociality of the helper has a positive impact on the recipient, it could improve the helper's levels of SWB by increasing positive emotions (the helping model; Midlarsky, 1991), reducing negative moods (the negative‐state relief model; Cialdini et al., 1981), and increasing feelings of satisfaction (the warm‐glow theory; Andreoni, 1989). On the other hand, the broaden‐and‐build theory argues that positive emotions, such as happiness and satisfaction, encourage individuals to show more prosocial tendencies (Fredrickson, 2001). Numerous empirical research supports this positive feedback loop (Aknin et al., 2012; Hui, 2022; Thoits & Hewitt, 2001; Xiong et al., 2023). For instance, individuals with greater SWB dedicated more time to volunteer work one year later; conversely, individuals who spent more time volunteering reported higher levels of SWB one year later (Thoits & Hewitt, 2001). Similarly, participants who recalled a prosocial experience reported more SWB in the moment, and later they tended to exhibit prosocial tendencies (Aknin et al., 2012; Lin et al., 2019). Thus, SWB and prosocial tendency would be connected in the network.

## THE UNDERLYING BRIDGE OF THE NETWORK: MEANING IN LIFE

Meaning in life refers to “the sense made of, and significance felt regarding, the nature of one's being and existence” (Steger et al., 2006). Over the past decade, meaning in life has been recognized as including two distinct dimensions: POM and SFM. POM involves the perception of meaning and purpose in life, while SFM involves the drive to pursue and achieve meaning and purpose. Later, researchers suggested that POM and SFM may be two separate concepts rather than interrelated dimensions of meaning in life (Chan, 2016; Liu & Gan, 2010; Steger, 2012).

Awe might be an antecedent of meaning in life, including of POM and SFM. Meaning in life can stem from self‐transcendent experiences, such as exposure to natural environments (Howell et al., 2013). Coincidentally, awe is the most common and profound emotion experienced in natural environments. Recent research supports this association. Dai et al. (2022) found that awe increased POM overall, mainly by motivating purpose pursuit. Rivera et al. (2020) found that awe diminished an individual's sense of significance, thereby motivating them to pursue more meaning (i.e., SFM) to satisfy the need for meaning. Additionally, awe motivates individuals towards self‐transcendence, leading them to pursue spiritual beliefs and the authentic‐self (Jiang & Sedikides, 2022; Van Cappellen & Saroglou, 2012). In this case, several empirical studies have found that trait awe can positively predict overall meaning in life (Zhao et al., 2019; Zhao & Zhang, 2022), as well as the separate dimension, POM (Fu et al., 2022). Although the relationship between trait awe and SFM is unclear, we assume that individuals with higher trait awe are more likely to be self‐transcendent and therefore pursue meaning in their lives. Therefore, we propose our third hypothesis:

Hypothesis 3 Trait awe is positively connected with both aspects of meaning in life, namely POM and SFM, in the network.

There is a complex relationship between meaning in life and SWB and prosociality. On the one hand, POM has been found to stably predict greater SWB and prosocial tendency. The meaning maintenance model (Heine et al., 2006) highlights that individuals' perception of meaning contributes directly to their SWB. Moreover, self‐determination theory also proposes that perceived meaning functions as an intrinsic sense of purpose, self‐regulation, and motivation that helps individuals cope with the challenges of life, thereby enhancing positive affect and life satisfaction. Meanwhile, POM also encourages individuals to autonomously engage in activities that contribute to personal survival and thriving, especially prosocial behaviors (Deci & Ryan, 2008). On the other hand, the relationships between SFM and SWB and prosocial tendency are debatable. SFM is regarded as a coping strategy to compensate for meaning when the existing meaning is threatened (Chang et al., 2021; Heine et al., 2006). In this case, SFM indeed helps individuals protect themselves from the negative effects of adverse events on SWB (Park, 2010). Moreover, individuals are more likely to engage in prosocial behaviors during the process of actively seeking meaning (Skaggs & Barron, 2006). However, for individuals whose meaning is not threatened, the strong motivation to SFM is seen as a loss of purpose in life and may be inappropriate or even detrimental for them. Several empirical studies have demonstrated these relationships. For example, POM has been found to predict positive psychological outcomes, such as greater life satisfaction, more happiness (Li et al., 2021; Soucase et al., 2023), and more prosocial tendencies (Fu et al., 2022; Wang et al., 2018; Xie et al., 2023). However, SFM typically predicts less SWB, while it predicts more SWB only for those with higher POM (Cohen & Cairns, 2012). Similarly, SFM motivated by death anxiety indeed predicts greater prosocial behaviors, which helps individuals maintain meaning (Chang et al., 2021). Nevertheless, for those whose meaning is not threatened, the positive relationship is not observed (Xie et al., 2023). Taken together, compared with SFM, POM may be more strongly associated with greater SWB and prosocial tendency.

Furthermore, meaning in life has been identified as a mediator in the relationships between trait awe and SWB (Zhao et al., 2019; Zhao & Zhang, 2022) and prosocial tendency (Fu et al., 2022). However, the specific mediating roles of POM and SFM remain unclear. In this study, we posit that POM could better explain these relationships than SFM. Consequently, within the entire network, POM may play a more crucial role than SFM in revealing the internal mechanisms of network functioning. Drawing inspiration from the concept of “bridges” in network analysis, we hypothesized that POM may function as the strongest bridge node within the network of traits awe, SWB, and prosocial tendency. Specifically, POM could link more different variables (i.e., bridge betweenness) and transmit the most impacts (i.e., bridge strength/expected influence) through the shortest pathway (i.e., bridge closeness) in the network. In sum, the fourth hypothesis posited is:

Hypothesis 4 POM is the strongest bridge node in the network.

## THE PRESENT STUDY

To test the four hypotheses, using network analysis, we constructed a network of trait awe, meaning in life (including POM and SFM), SWB (including subjective happiness and life satisfaction), and prosocial tendency (including willingness to donate money and volunteer time). First, to examine H1–H3, we focused on the direct connections between trait awe, SWB, and prosocial tendency, as well as on indirect connections “mediated” by meaning in life. Second, we examined differences in the edge weights for specific connections, addressing questions such as whether trait awe is equally associated with POM and SFM, and whether POM is more strongly associated with SWB and prosocial tendency. These explorations aided in comparing the local roles of POM and SFM in the aforementioned indirect connections, laying the foundation for further exploration of their bridging roles in the overall network. Third, to test H4, we assessed the overall bridging roles of POM and SFM within the network using bridge centralities (e.g., bridge strength/expected influence, bridge betweenness, and bridge closeness). Finally, we evaluated the accuracy of the network and the stability of the bridge centralities. Additionally, a mediation analysis was conducted to examine the mediating roles of POM and SFM between trait awe, SWB, and prosocial tendency (see Supporting Information**)**.

## METHOD

### Participants

The current study was approved by the Ethics Committee of Beijing Normal University (reference number: 202304250080). For network analysis, it is recommended to have a sample size that is at least 10 times larger than the number of parameters estimated. We recruited 575 Chinese adults using Credamo software (https://www.credamo.com) in May 2023. A total of 37 participants were excluded for failing an attention check. The final valid sample included 538 participants (252 males, 286 females; *M*_age_ = 19.86, *SD*_age_ = 1.51) for a 93.57% effective rate.

### Procedures

After signing the informed consent form, participants completed a series of measurements of trait awe, meaning in life, SWB, and prosocial tendency. The order of each scale was randomly arranged, and the items within each scale were also randomized. Following the survey, participants were thanked and asked for any additional comments.

### Measures

#### Trait awe

Trait awe was assessed using the awe subscale from the Dispositional Positive Emotions Scale (Shiota et al., 2006). Participants were asked to report their agreement with six items (e.g., “*I often feel awe*”) on a 7‐point Likert scale (1 = *extremely disagree*, 7 = *strongly agree*). Higher scores indicated a higher level of trait awe. Cronbach's alpha was .82.

#### Meaning in life

The 9‐item Chinese version of the Meaning in Life Questionnaire (MLQ; Liu & Gan, 2010) measured the two aspects of meaning: POM (MLQ‐P; e.g., “*I understand my life's meaning*”) and SFM (MLQ‐S; e.g., “*My life has no clear purpose*[R]”). Participants rated their agreement on a 7‐point Likert scale (1 = *absolutely untrue*, 7 = *absolutely true*). Cronbach's alphas for MLQ‐P and MIQ‐S were .83 and .91, respectively.

#### SWB

SWB, including the two aspects of subjective happiness and life satisfaction, was measured using the Well‐Being Index Scale (Campbell et al., 1976) and Satisfaction with Life Scale (Diener et al., 1985). For subjective happiness, participants reported their level of eight semantic differential emotions on a 7‐point Likert scale (e.g., “*boring‐interesting*”; 1 = *boring*, 7 = *interesting*); Cronbach's alpha was .91. For life satisfaction, participants rated their agreement with five items (e.g., “*I am satisfied with my life*”) on a 7‐point Likert scale (1 = *extremely disagree*, 7 = *extremely agree*); Cronbach's alpha was .85.

#### Prosocial tendency

Prosocial tendency was assessed by four items adapted from Rudd et al. (2012), evaluating participant's willingness to volunteer time and donate money to help a charity (e.g., “*How willing are you to donate your money/volunteer your time to support a worthy cause?*”) on a 7‐point Likert scale (1 = *not at all*, 7 = *very*). Cronbach's alphas were .86 (donate money) and .76 (volunteer time).

#### Data analysis

We first used Jamovi (an open statistical software package) for description statistics for trait awe, meaning in life, SWB, and prosocial tendency. Then, we further conducted a series of network analyses using the R Statistical Software (v4.3.1; R Core Team, 2023), as follows: (1) estimate the network structure to visualize the basic associations between nodes; (2) examine differences in edge weights; (3) test bridge centralities, including bridge strength, bridge betweenness, and bridge closeness, to identify the bridge function of meaning in life in the network; (4) test the accuracy and stability of the network. Detailed analysis methods, parameter indicators, packages used, and additional tables and figures can be found in the Supporting Information.

## RESULTS

### Descriptive statistics

The means, standard deviations, skewness, and kurtosis of all variables measured are presented in Table 1.

**TABLE 1:** Description statistics of trait awe, meaning in life, SWB, and prosocial tendency.

Node	Description statistics			
	*M*	*SD*	Skewness	Kurtosis
TA: Trait Awe	4.08	1.06	−0.07	0.35
Meaning in Life (MIL)				
MIL1: POM	4.39	1.14	−0.10	0.32
MIL2: SFM	4.95	1.19	−0.31	0.42
Subjective Well‐Being (SWB)				
SWB1: Subjective happiness	4.80	1.18	−0.08	−0.07
SWB2: Life satisfaction	3.97	1.13	0.08	0.42
Prosocial Tendency (PT)				
PT1: Donating money	4.43	1.38	−0.44	0.31
PT2: Volunteering time	4.66	1.30	−0.42	0.45

Abbreviations: *M*, mean; *SD*, standard deviation.

### Network structures

**FIGURE 1:**
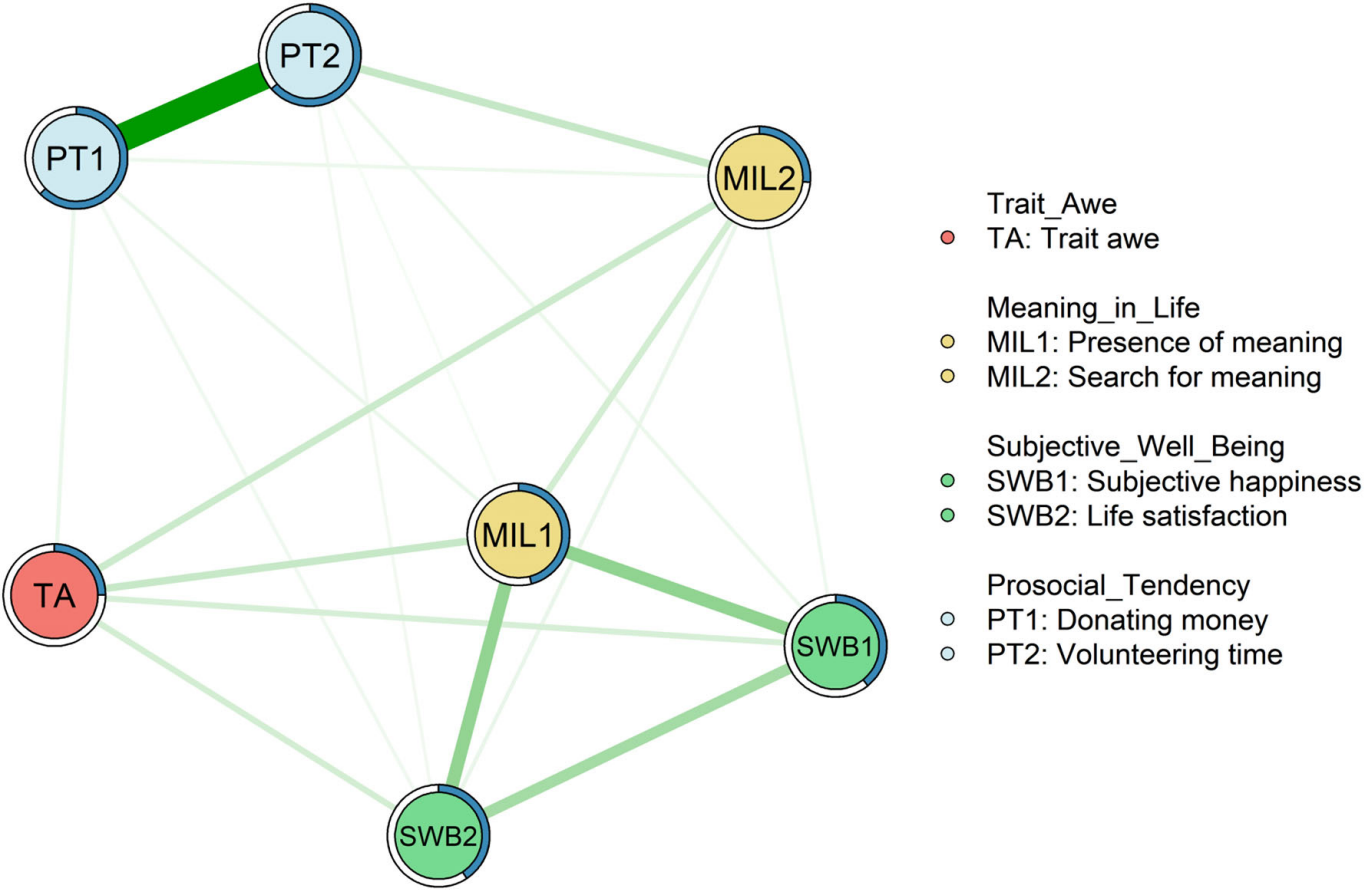
The network of Trait Awe, Meaning in Life, Subjective Well‐Being, and Prosocial Tendency (*n* = 538). The thickness and darkness of lines indicate the weight of each edge. Green edges represent a positive correlation, and red edges represent a negative correlation.

The estimated network is shown in Figure 1. There are seven nodes and 21 edges [7 × (7 – 1)/2] in the network, which includes 20 (95.24%) non‐zero weights (see full edge weights in Table 2). This signifies close interconnections between trait awe, meaning in life (POM and SFM), SWB (subjective happiness and life satisfaction), and prosocial tendency (willingness to donate money and volunteer time).

**TABLE 2:** The weight matrix and centrality of the network.

Node	Weight matrix							Standardized bridge centrality		
	TA	MIL1	MIL2	SWB1	SWB2	PT1	PT2	Bridge strength	Bridge Betweenness	Bridge closeness
TA: Trait Awe	—	0.154	0.142	0.114	0.122	0.059	0	0.45	−0.78	−0.14
Meaning in Life (MIL)										
MIL1: POM	0.154	—	0.127	0.308	0.302	0.053	0.028	1.65	0.94	1.82
MIL2: SFM	0.142	0.127	—	0.045	0.061	0.052	0.159	−0.18	1.51	0.14
Subjective Well‐Being (SWB)										
SWB1: Subjective happiness	0.114	0.308	0.045	—	0.252	0.003	0.052	0.12	−0.78	0.21
SWB2: Life satisfaction	0.122	0.302	0.061	0.252	—	0.044	0.040	0.34	−0.78	0.22
Prosocial Tendency (PT)										
PT1: Donating money	0.059	0.053	0.052	0.003	0.044	—	0.713	−1.36	−0.78	−1.27
PT2: Volunteering time	0	0.028	0.159	0.052	0.040	0.713	—	−1.03	0.65	−0.97

In the network, “trait awe” first exhibits positive associations with dimensions of SWB such as “subjective happiness” (TA‐SWB1: 0.114) and “life satisfaction” (TA‐SWB2: 0.122). Notably, “trait awe” is indirectly and positively connected to “subjective happiness” and “life satisfaction” through “POM” (TA‐MIL1: 0.154, MIL1‐SWB1/SWB2: 0.308/0.302) and/or “SFM” (TA‐MIL2: 0.142, MIL2‐SWB1/SWB2: 0.045/0.061).

Second, “trait awe” has a direct positive connection with “donating money” (TA‐PT1: 0.059), but not with “volunteering time” (TA‐PT2: 0). Crucially, trait awe is indirectly connected positively with “donating money” and “volunteering time” through “POM” (MIL1‐PT1/PT2: 0.053/0.028) and/or “SFM” (MIL2‐PT1/PT2: 0.052/0.159). Additionally, dimensions of SWB and prosocial tendency are also connected. Both “subjective happiness” and “life satisfaction” are directly and positively connected with “donating money” (SWB1/SWB2‐PT1: 0.003/0.044) and “volunteering time” (SWB1/SWB2‐PT2: 0.052/0.040).

Furthermore, the bootstrapped difference test for edge weights (see Figure S1) revealed some differences between specific connections. First, “POM” is more strongly associated with “subjective happiness” and “life satisfaction” (MIL1‐SWB1/SWB2: 0.308/0.302) than “SFM” (MIL2‐SWB1/SWB2: 0.045/0.061; represented as black boxes). Second, “SFM” and “POM” show similar connections with “donating money” (MIL1/MIL2‐PT1: 0.052/0.053; represented as gray boxes), while “SFM” exhibits more connections with “volunteering time” than “POM” (MIL1/MIL2‐PT2: 0.028/0.159; represented as black boxes). Moreover, no significant differences were found in the specific relationships between trait awe and meaning in life (i.e., TA‐MIL1, TA‐MIL2) or SWB (i.e., TA‐SWB1, TA‐SWB2; represented as gray boxes), or between SWB and prosocial tendency (i.e., SWB1‐PT1, SWB2‐PT1, SWB2‐PT1, SWB2‐PT2; represented as gray boxes).

### Network centrality

**FIGURE 2:**
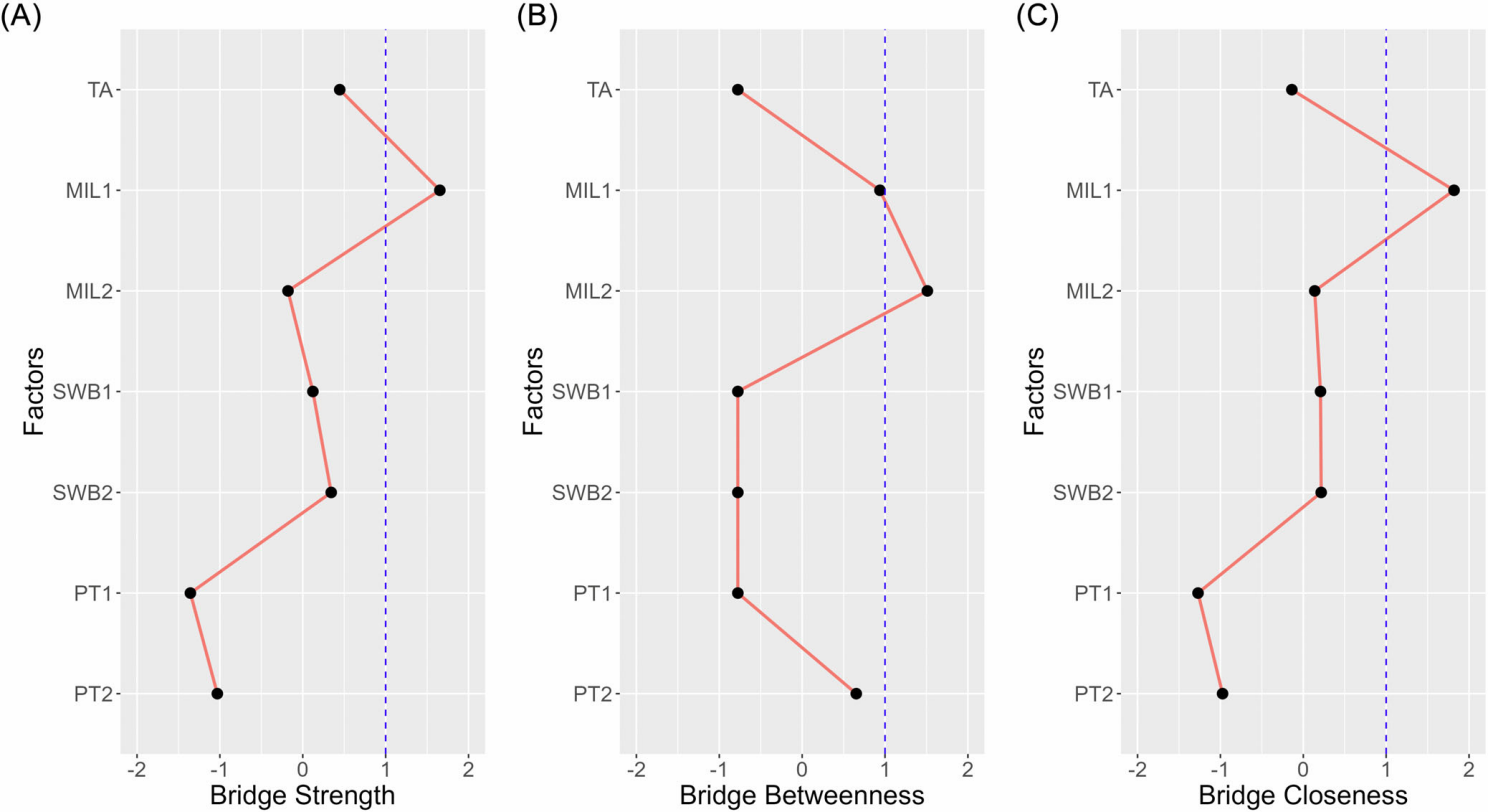
Standardized bridge centralities of the network. MIL1, presence of meaning (POM); MIL2, searching for meaning (SFM); PT1, donating money; PT2, volunteering time; SWB1, subjective happiness; SWB2, life satisfaction; TA, trait awe.

To identify the bridging node within the network, we estimated bridge centralities and conducted bootstrapped difference tests for bridge centralities (see Table 2, Figure 2, and Figure S2). As indicated, “POM” (MIL1) has the highest standardized bridge strength (1.65) and bridge closeness (1.82), which are significantly higher than those of other nodes in the network (represented as black boxes; see Figure S2). Moreover, “SFM” (MIL2) exhibits the highest standardized bridge betweenness (1.51). However, no significant difference is observed in the bridge betweenness among nodes in the network (represented as gray boxes), indicating that the order of bridge betweenness cannot be interpreted with confidence. In sum, POM was identified as the unique bridge with the strongest bridge strength and bridge closeness.

### Network accuracy and stability estimation

**FIGURE 3:**
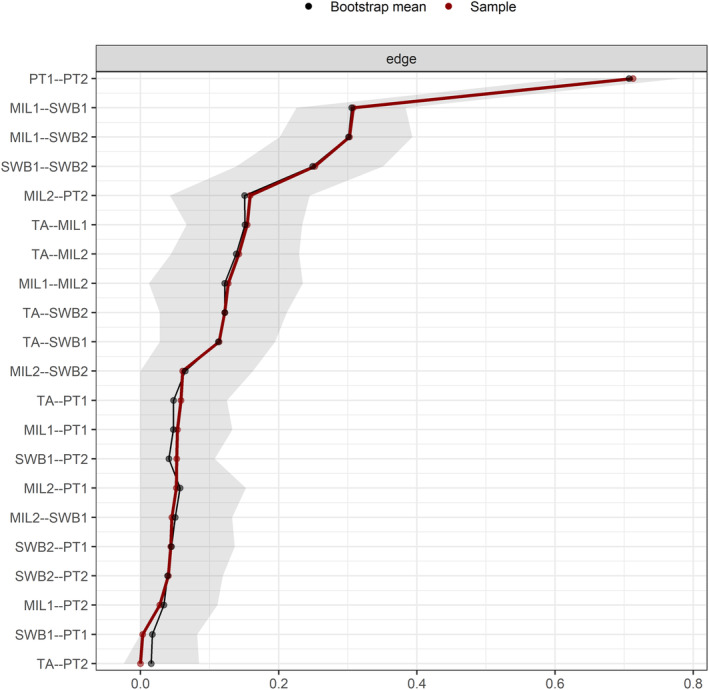
Nonparametric bootstrapped confidence intervals of estimated edges in the network. The red line indicates the estimated edge, and the dark area indicates the 95% bootstrap CI. MIL1, presence of meaning (POM); MIL2, searching for meaning (SFM); PT1, donating money; PT2, volunteering time; SWB1, subjective happiness; SWB2, life satisfaction; TA, trait awe.

**FIGURE 4:**
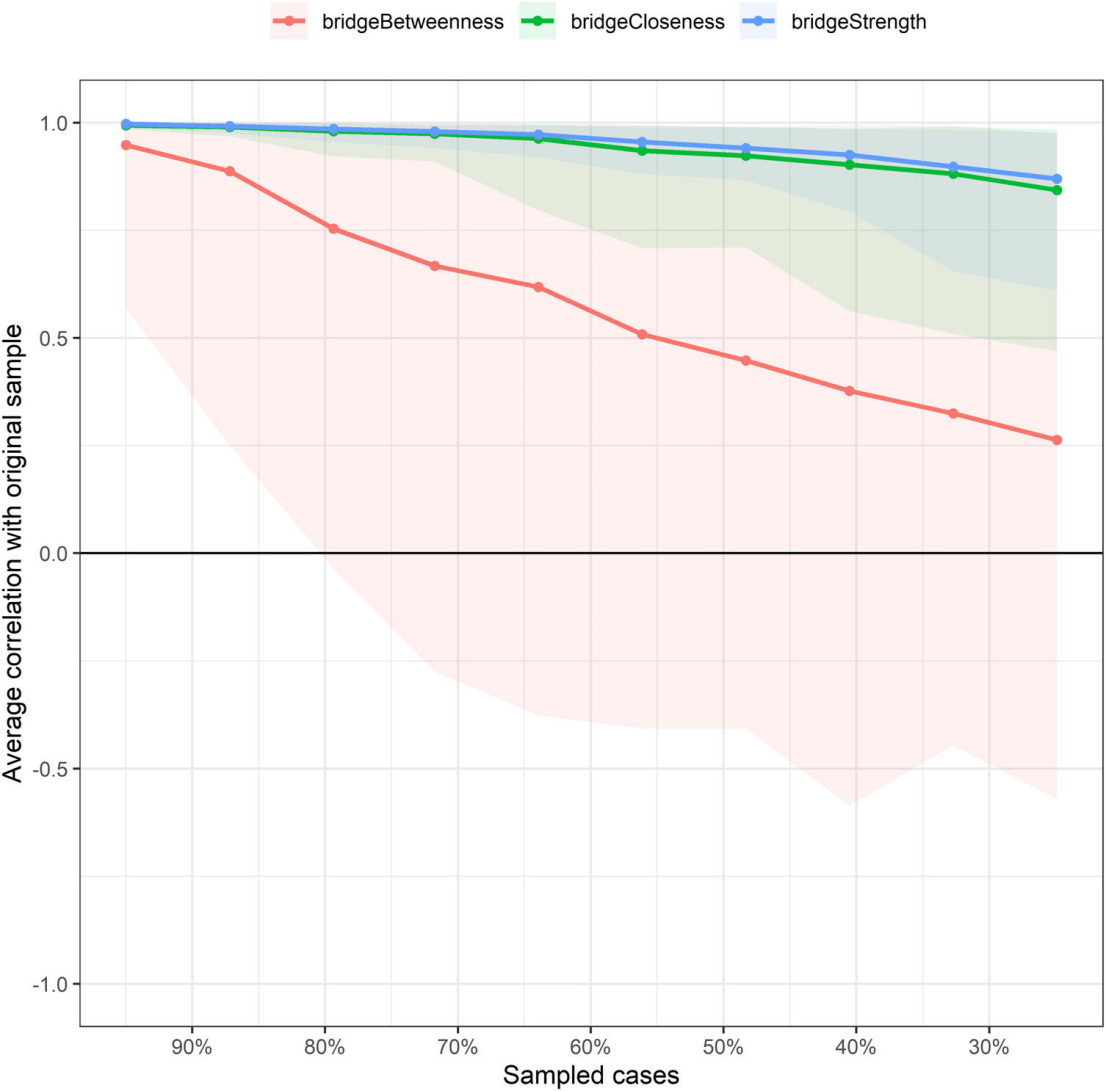
Average correlation with original sample after excluding a certain proportion of cases in the network. x‐axis: the percentage of cases of the original sample included at each step. y‐axis: average correlation between the original network's centrality indices and the re‐estimated network's centrality indices after excluding increasing percentages of cases. A value exceeding 0.25 is considered acceptable, while a value exceeding 0.50 is considered excellent.

The bootstrapped test initially revealed narrow 95% confidence intervals (CIs) of the edge weights, ensuring the accuracy of the network (see Figure 3). Subsequently, a case‐dropping bootstrap procedure (see Figure 4) demonstrated that, as the sample proportion decreases, the correlations between the bridge strength and bridge closeness of the new sample network and those of the original sample network weaken slightly, while the correlation for bridge betweenness centralities weakens significantly. Furthermore, the correlation stability (CS) coefficients for bridge strength (0.67) and bridge closeness (0.60) are above 0.50, but the CS coefficient for bridge betweenness is below 0.25. In conclusion, bridge strength and bridge closeness are highly stable, whereas the bridge betweenness is not.

## DISCUSSION

The present study, for the first time, employed network analysis to elucidate the complex relationships between trait awe, meaning in life (POM and SFM), SWB (subjective happiness and life satisfaction), and prosocial tendency (willingness to donate money and volunteer time), and to show how POM acts as a bridge connecting and influencing the entire network. These findings are pivotal for the advancement and enhancement of both subjective well‐being and prosocial tendencies.

### Network connections between trait awe, SWB, and prosocial tendency

Trait awe was positively connected with SWB in the network, supporting Hypothesis 1. Specifically, individuals who experienced awe more frequently and intensively tended to feel more positive emotions and be more satisfied with their lives. These positive associations are consistent with previous studies, further demonstrating the positive emotional and cognitive benefits of awe in individuals' lives (Dong & Ni, 2019; Zhao et al., 2019). More recently, researchers have examined whether awe buffered the negative events and increased SWB during the COVID‐19 pandemic (Braswell & Prichard, 2023; Büssing et al., 2021; Monroy et al., 2023). Büssing et al. (2021) indicated that awe may not be a panacea that directly buffers against adverse events in life and reduces burdens, but it does increase well‐being during difficult times. Later, researchers found that individuals who experienced more daily awe reported less stress and somatic health symptoms, and felt more well‐being (Monroy et al., 2023). Similarly, Braswell and Prichard (2023) found that individuals higher in trait awe tended to report higher resilience to COVID‐19, even when controlling for religiosity. In the post‐COVID‐19 era, awe might still be a valuable psychological resource for individuals to recover from the pandemic and enhance their happiness and life satisfaction.

Trait awe was positively related to the prosocial tendency to donate money rather than to volunteer time in the network, partially supporting Hypothesis 2. The positive relationship between trait awe and willingness to donate money is consistent with previous studies, indicating that awe could facilitate individuals' prosocial decisions involving donating money (Guan et al., 2019; Piff et al., 2015). However, although previous studies have shown that awe could encourage individuals to volunteer their time to help others (Guan et al., 2019; Rudd et al., 2012), we did not replicate this finding at the trait awe level. For one thing, the network analysis estimated the edge weight between nodes using partial correlation, which might weaken the association between trait awe and willingness to volunteer time. For another, the network analysis was data‐driven and therefore sensitive to slight changes in data. Moreover, the general prosocial nature of trait awe in various contexts (Fu et al., 2022; J.‐J. Li et al., 2019) did not lessen our confidence in the relationship between trait awe and willingness to volunteer time. Future studies should further examine this relationship using multiple methods. Additionally, SWB and prosocial tendency—two downstream aspects of trait awe—were also positively connected in the network. For the present cross‐sectional network, we argue that these findings fully support the positive feedback loop between SWB and prosocial tendency (Hui, 2022; Xiong et al., 2023). Specifically, individuals who experience more positive affects and have greater life satisfaction are more likely to exhibit prosocial tendencies by donating money and volunteering their time. Conversely, those with higher prosocial tendencies are more likely to experience more positive affects and higher life satisfaction. More importantly, our network suggests that trait awe acts as an external trigger, continuously contributing energy to the positive feedback loop between SWB and prosocial tendency.

### Network indirect connections through meaning in life

In the present study, we also focused on the indirect connections between trait awe, SWB, and prosocial tendency through meaning in life. Both POM and SFM could link trait awe to SWB and prosocial tendency, shedding light on why trait awe is positively related to SWB and prosocial tendency. As an antecedent, trait awe was equally associated with POM and SFM, supporting Hypothesis 3. Consistent with Fu et al. (2022), we found that individuals higher in trait awe are more likely to perceive their current lives as meaningful. In addition, we have provided the first empirical evidence that they are also more likely to actively pursue more meaning.

In terms of downstream effects, meaning in life was related to SWB and prosocial tendency. First, in the network, both POM and SFM were positively connected to subjective happiness and life satisfaction, with POM having stronger connections. In other words, compared with SFM, POM has a greater impact on subjective happiness and life satisfaction in the network. These findings support that POM can better predict more SWB than SFM (J.‐B. Li et al., 2021). POM might also act as a protective factor against the negative outcomes of SFM on SWB (Cohen & Cairns, 2012; N. Park et al., 2010). Therefore, compared with SFM, POM may better explain the indirect relationship between trait awe and SWB.

Second, both POM and SFM were positively associated with the prosocial tendency to donate money and volunteer time, providing support for previous studies (Chang et al., 2021; Fu et al., 2022). Notably, we further observed that SFM showed a stronger positive association with the tendency to volunteer time than POM. In other words, individuals with higher SFM are equally likely to engage in donation but more likely to engage in volunteering compared with those with higher POM. This is possibly because individuals searching for meaning are inclined to perform costly prosocial behaviors, which are seen as sources of meaning. Actions that require effort or other costs are endowed with greater value and meaning (Olivola & Shafir, 2013). Driven by a strong desire to search for meaning, individuals tend to engage in behaviors that are both costly for themselves and beneficial for others (Dakin et al., 2021). However, volunteering one's time appears to be more costly and meaningful than donating money. As a result, individuals with higher SFM exhibited a similar willingness to donate money but a greater willingness to volunteer time compared with those with higher POM. In sum, both POM and SFM may explain the indirect relationship between trait awe and prosocial tendency, with SFM more effectively explaining the indirect relationship between trait awe and the willingness to volunteer time.

### Bridge node of the network: POM

While we initially compared the roles of POM and SFM in the local network, we still need to examine their overall bridging roles in the global network. The roles of POM and SFM in the local network determine their roles in the overall network. First, POM stably exhibited the largest bridge strength among all nodes, indicating the strongest connectedness with different variables in the network. This suggests that POM maximally transmits the influence of trait awe to SWB and prosocial tendency, particularly regarding SWB. In contrast, SFM exhibited relatively weaker connections to certain nodes, such as subjective happiness and life satisfaction, reducing its overall influence in the network. Second, SFM showed the highest bridge betweenness, but this result lacked distinctiveness and stability. Therefore, we conservatively argue that there is no significant “mediator” connecting the entire network. Third, POM also exhibited the highest bridge closeness, signifying the shortest average distance from other variables in the network. This enables POM to efficiently transmit influences from trait awe to dimensions of SWB and prosocial tendency. In comparison, SFM transmits influence at a lower rate as it is situated further away from the other variables. Overall, considering both bridge strength and closeness, our results essentially support Hypothesis 4, suggesting that POM acts as the key bridge node within the network. POM efficiently transmits the impacts of trait awe on SWB (Zhao et al., 2019) and prosocial tendency (Fu et al., 2022). However, it is crucial to note that the role of bridges pertains to the entire network, not to specific local networks. While POM is recognized as a bridge in the present network, the pivotal role of SFM in the local link between trait awe and prosocial tendency cannot be disregarded.

### Implications

The current work has some important theoretical and practical implications. To our knowledge, our study is the first to use a network analysis approach to examine the complex relationships among trait awe, SWB, and prosocial tendency and to explore meaning in life as an explanatory mechanism. Visualizing all relevant dimensions in a comprehensive network provides a clear understanding of the intricate relationships among trait awe, meaning in life, SWB, and prosocial tendency. This enhances our insight into the underlying mechanisms, particularly the specific role of POM. Network analysis compensates for the shortcomings of factor models to a certain extent. Moreover, our study replicates previous findings using a novel network approach, thereby confirming the accuracy and robustness of previous conclusions and providing valuable insights and inspiration for future research on a similar topic.

In terms of practical implications, the nodes with the highest bridge strength play crucial roles in activating or inhibiting other nodes between different variables (or communities). Enhancing these central nodes might improve the overall connectivity of the network. Meanwhile, nodes with the highest bridge closeness play pivotal roles in connecting other nodes and facilitating the transmission of positive or negative influences within the network. These nodes are considered as important targets for prevention and intervention efforts. In our study, regardless of bridge strength or bridge closeness, POM consistently exhibits the highest values. Therefore, improving POM could be a potential strategy for increasing SWB and prosocial tendencies. Specifically, individuals are encouraged to identify and reflect on the core values, develop a profound understanding of the meaning and purpose of life, and search for more meaning and purpose to maintain perceived meaning. Improvements in meaning in life might contribute to experiencing greater happiness and inspiring more prosocial behavior.

### Limitations and future directions

Our study has several limitations that warrant further investigation. First, in the network analysis, all variables were placed at the same level, with neither independent nor dependent variables identified. Moreover, the present cross‐sectional network only provides weights for edges and not the directions of the edges. Although interpretations and conclusions drawn from this network are based on existing theories and empirical evidence rather than on data‐driven results, caution is warranted when interpreting the causality between antecedent and downstream variables in the network. Future studies should employ a cross‐lagged panel network analysis to investigate the specific directions of edges and to determine the causal relationships between these variables. Second, by considering the magnitudes of edge weights, we compared the respective roles of POM and SFM in the indirect relationships between trait awe and SWB and prosocial tendency. This approach is somewhat incomplete and controversial. Future research could further address this issue by, for example, comparing the distances of different paths through POM and SFM to determine which path is shorter and more appropriate for impact transmission (Bringmann et al., 2019). This approach is helpful for understanding how the influence is transmitted between two nodes in the networks. Third, although we found a positive relationship between trait awe and SFM, this remains to be explored in future empirical studies. Additionally, owing to the debate regarding the relationship between POM and SFM (Steger, 2012), future research is recommended to utilize multiple measures to further examine the relationships between trait awe and dimensions of meaning.

## CONCLUSION

Using network analysis, this study constructed a network of trait awe, meaning in life (i.e., POM and SFM), SWB (i.e., subjective happiness and life satisfaction), and prosocial tendency (i.e., willingness to donate money and volunteer time) and identified the bridging role of meaning in life within the network. In this network, trait awe exhibited direct and indirect connections to dimensions of SWB and prosocial tendency. POM and SFM played similar but distinct “mediating” roles in the indirect connections. Furthermore, POM was identified as a bridge node that could efficiently transmit the impacts within the entire network. Overall, the present study sheds light on the underlying mechanisms of the relationships among trait awe, SWB, and prosocial tendency, providing insights for improving SWB and fostering prosocial tendencies.

## CONFLICT OF INTEREST STATEMENT

The authors report no potential conflicts of interest.

## ETHICS STATEMENT

The current study was approved by the Ethics Committee of Beijing Normal University (Reference number: 202304250080).

## Supporting information


**Table S1:** The correlations between trait awe, meaning in life, subjective well‐being, and prosocial tendency.
**Table S2.** The mediation of meaning in life between trait awe and subjective well‐being and prosocial tendency.
**Figure S1.** Bootstrapped difference test for edge‐weights in the network.
**Figure S2.** Bootstrapped difference test for bridge centralities in the network.
**Figure S3.** The mediation of meaning in life between trait awe, subjective well‐being, and prosocial tendency.
