# Supplementary material for: The unique role of meaning in life in the relationships between trait awe, subjective well‐being, and prosocial tendency: A network analysis
Source: Psych J. 2024 Feb 16;13(4):575–87. doi: 10.1002/pchj.733 (PMC11317181; doi:10.1002/pchj.733)
Supplement: Supplementary file 1 — Table S1: The correlations between trait awe, meaning in life, subjective well‐being, and prosocial tendency. Table S2. The mediation of meaning in life between trait awe and subjective well‐being and prosocial tendency. Figure S1. Bootstrapped difference test for edge‐weights in the network. Figure S2. Bootstrapped difference test for bridge centralities in the network. Figure S3. The mediation of meaning in life between trait awe, subjective well‐being, and prosocial tendency. [file PCHJ-13-575-s001.docx]

**The Unique Role of Meaning in Life in the Relationships Between Trait Awe, Subjective Well-Being, and Prosocial Tendency: A Network Analysis**

**Supplementary Materials**

***Data Analysis***

Firstly, we firstly used jamovi (an open-source statistical software) to conduct descriptive statistics for trait awe, meaning in life (presence of and search for meaning, abbreviated as POM and SFM), SWB (subjective happiness and life satisfaction), and prosocial tendency (willingness to donate money and volunteer time).

Subsequently, we utilized the *qgraph* package (Epskamp et al., 2012) in the R program to estimate the network. Each factor mentioned above was set as a “node,” and connections between these factors were depicted s “edges”. Notably, the thickness of edges represented the strength of relationships between nodes, with color indicating whether nodes were positively correlated (green) or negatively correlated (red; Epskamp et al., 2012). We employed Extended Bayesian Information Criterion (EBIC) and graphical least absolute shrinkage and selection operator (GLASSO) network models to construct a sparse network model, which could be helpful to reduce the possibility of overfitting and better to understand for readers. Following edge-weight estimation, we utilized the *bootnet* package (Epskamp et al., 2018) and performed bootstrapped difference tests on edge-weights to identify significant differences.

Furthermore, we utilized the *bridge* function to estimate the bridge centrality parameters, such as bridge strength/expected influence, bridge betweenness, and bridge closeness (Opsahl et al., 2010). As all edges in the current network had positive weights, we used the bridge strength rather than bridge expected influence. Bridge strength sums the absolute values of weights on the edges connecting a node to others in different communities, measuring the overall connectedness, influence, or importance of a node between communities. Higher bridge strength indicates more influence the node can transmit between communities. Bridge betweenness calculates how often a node lies on the shortest path between nodes in different communities, revealing potential mediators in network transactions. Bridge closeness assesses a node’s average proximity, in terms of edge distance, to other nodes in different communities. Stronger the bridging closeness implies shorter average distances and faster transmission between different communities. We focused on the node with the highest centrality or multiple nodes whose centrality exceeds 1. To test uniqueness of each node, we utilized the *bootnet* package and performed the bootstrapped difference tests on bridge centrality to find out whether the node significantly differs from other nodes in the bridge centrality.

Finally, we used the *bootnet* package to assess the accuracy and stability of the estimated network (Epskamp et al., 2018). Edge-weight accuracy was evaluated through bootstrap analysis of 95% confidence intervals (CIs). Greater overlap between estimated and bootstrapped edges, as well as narrower 95% bootstrap CI, indicates higher accuracy. Centrality stability was assessed using a case-dropping bootstrap, with a centrality stability coefficient exceeding 0.25 considered acceptable and exceeding 0.5 considered excellent. Moreover, as the sample proportion decreases, the correlation between the bridge centrality of the network in the new sample and that of the original sample decreases. The smaller the decrease, the more stable the bridge centrality.

Additionally, we performed some supplementary analyses in SPSS, including correlation analyses among trait awe, meaning in life, SWB, and prosocial tendency, and mediation analysis of meaning in life in the relationships between trait awe, SWB, and prosocial tendency using process package (Hayes, 2013).

**Table S1: The Correlations Between Trait Awe, Meaning in Life, Subjective Well-Being, and Prosocial Tendency**

| **Node** | **TA** | | **MIL1** | | **MIL2** | | **SWB1** | | **SWB2** | | **PT1** | | **PT2** | |
| --- | --- | --- | --- | --- | --- | --- | --- | --- | --- | --- | --- | --- | --- | --- |
| **TA: Trait Awe** | — |  |  |  |  |  |  |  |  |  |  |  |  |  |
| **Meaning in Life (MIL)** |  |  |  |  |  |  |  |  |  |  |  |  |  |  |
| MIL1: POM | 0.42 | *** | — |  |  |  |  |  |  |  |  |  |  |  |
| MIL2: SFM | 0.33 | *** | 0.38 | *** | — |  |  |  |  |  |  |  |  |  |
| **Subjective Well-Being (SWB)** |  |  |  |  |  |  |  |  |  |  |  |  |  |  |
| SWB1: Subjective happiness | 0.37 | *** | 0.56 | *** | 0.32 | *** | — |  |  |  |  |  |  |  |
| SWB2: Life Satisfaction | 0.38 | *** | 0.56 | *** | 0.33 | *** | 0.52 | *** | — |  |  |  |  |  |
| **Prosocial Tendency** |  |  |  |  |  |  |  |  |  |  |  |  |  |  |
| PT1: Donating money | 0.29 | *** | 0.36 | *** | 0.38 | *** | 0.31 | *** | 0.34 | *** | — |  |  |  |
| PT2: Volunteering time | 0.28 | *** | 0.36 | *** | 0.42 | *** | 0.32 | *** | 0.34 | *** | 0.78 | *** | — |  |

**Table S2: The Mediation of Meaning in Life Between Trait Awe and Subjective Well-Being and Prosocial Tendency**

| **Variable** |  | Indirect paths |  | **Estimate** | | ***SE*** | | **95% Confidence Interval** | | | |
| --- | --- | --- | --- | --- | --- | --- | --- | --- | --- | --- | --- |
|  |  |  |  |  |  |  |  | **Lower** | | **Upper** | |
| **SWB1** |  | IND1: TA → MIL1 → SWB1 |  | .19 |  | .03 |  | .14 |  | .25 |  |
|  |  | IND2: TA → MIL2 → SWB1 |  | .02 |  | .01 |  | .0004 |  | .04 |  |
|  |  | IND3: TA → MIL1 → MIL2 →SWB1 |  | .01 |  | .01 |  | .0002 |  | .02 |  |
|  |  | C1: IND1 – IND2 |  | .17 |  | .03 |  | .12 |  | .24 |  |
|  |  | C2: IND1 – IND3 |  | .18 |  | .03 |  | .13 |  | .24 |  |
|  |  | C3: IND2 – IND3 |  | .01 |  | .01 |  | -.003 |  | .03 |  |
| **SWB2** |  | IND1: TA → MIL1 → SWB1 |  | .19 |  | .03 |  | .14 |  | .25 |  |
|  |  | IND2: TA → MIL2 → SWB1 |  | .02 |  | .01 |  | .002 |  | .05 |  |
|  |  | IND3: TA → MIL1 → MIL2 →SWB1 |  | .01 |  | .01 |  | .001 |  | .03 |  |
|  |  | C1: IND1 – IND2 |  | .17 |  | .03 |  | .11 |  | .23 |  |
|  |  | C2: IND1 – IND3 |  | .18 |  | .03 |  | .12 |  | .24 |  |
|  |  | C3: IND2 – IND3 |  | .01 |  | .01 |  | -.005 |  | .03 |  |
| **PT1** |  | IND1: TA → MIL1 → SWB1 |  | .09 |  | .03 |  | .14 |  | .14 |  |
|  |  | IND2: TA → MIL2 → SWB1 |  | .06 |  | .02 |  | .03 |  | .10 |  |
|  |  | IND3: TA → MIL1 → MIL2 →SWB1 |  | .03 |  | .01 |  | .02 |  | .06 |  |
|  |  | C1: IND1 – IND2 |  | .03 |  | .04 |  | -.04 |  | .10 |  |
|  |  | C2: IND1 – IND3 |  | .06 |  | .03 |  | -.01 |  | .11 |  |
|  |  | C3: IND2 – IND3 |  | .02 |  | .02 |  | -.01 |  | .06 |  |
| **PT2** |  | IND1: TA → MIL1 → SWB1 |  | .09 |  | .02 |  | .04 |  | .13 |  |
|  |  | IND2: TA → MIL2 → SWB1 |  | .06 |  | .02 |  | .03 |  | .11 |  |
|  |  | IND3: TA → MIL1 → MIL2 →SWB1 |  | .04 |  | .01 |  | .02 |  | .06 |  |
|  |  | C1: IND1 – IND2 |  | .02 |  | .03 |  | -.05 |  | .09 |  |
|  |  | C2: IND1 – IND3 |  | .05 |  | .03 |  | -.01 |  | .10 |  |
|  |  | C3: IND2 – IND3 |  | .03 |  | .02 |  | -.01 |  | .07 |  |

***Note.*** Mediation model is shown in Figure S3; Bootstrap samples: N = 5000; TA = trait awe, MIL1 = POM, MIL2 = SFM, SWB1 = subjective happiness, SWB2 = life satisfaction, PT1 = donating money, PT2 = volunteering time


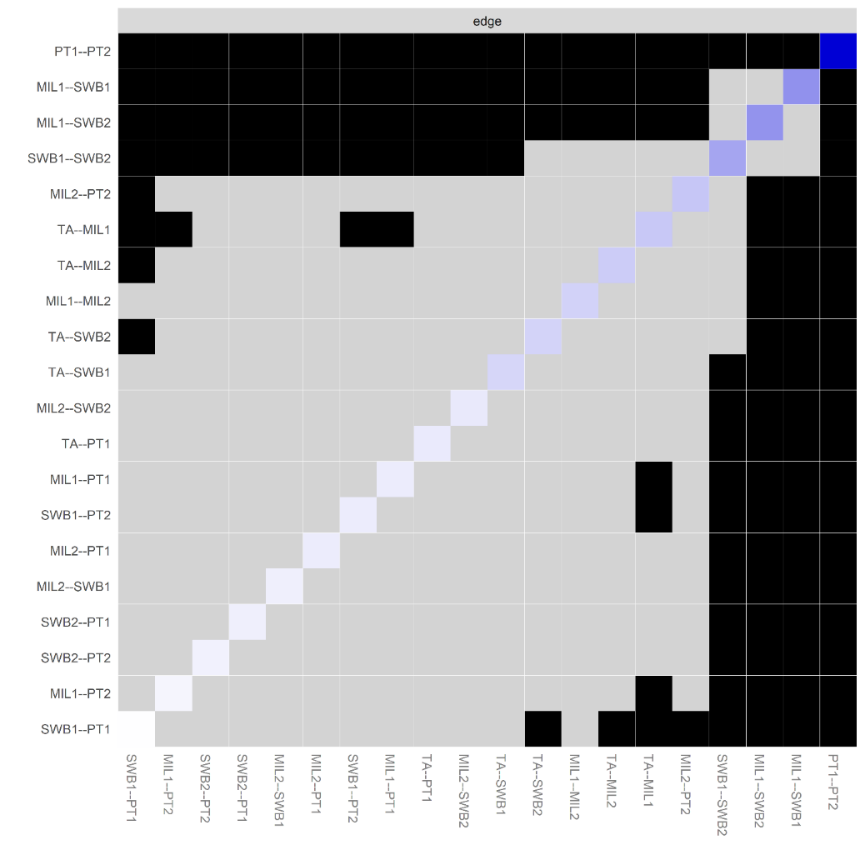


**Figure S1.** **Bootstrapped Difference Test for Edge-Weights in the Network.**

***Note*.** TA, trait awe; MIL1, POM; MIL2, SFM; SWB1, subjective happiness; SWB2, life satisfaction; PT1, donating money; PT2, volunteering time. The color of the boxes indicates whether the edge-weight differs significantly from each other (i.e., black) or do not differ significantly (i.e., grey). The diagonal line displays edge weight strength, ranging from strong negative associations (red), weak associations (white), and strong positive associations (blue).


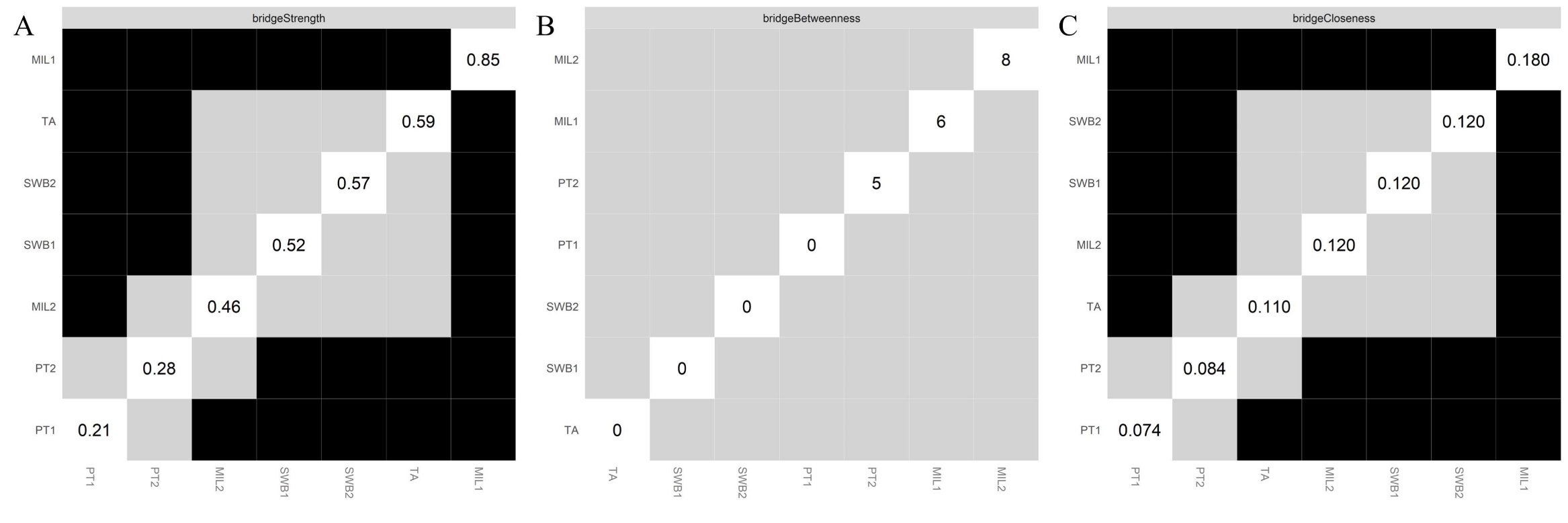


**Figure S2. Bootstrapped Difference Test for Bridge Centralities in the Network.**

***Note*.** TA, trait awe; MIL1, POM; MIL2, SFM; SWB1, subjective happiness; SWB2, life satisfaction; PT1, donating money; PT2, volunteering time. The color of the boxes indicates whether the bridge centrality differs significantly from each other (i.e., black) or do not differ significantly (i.e., grey). The diagonal line displays the unstandardized bridge centralities of each node.


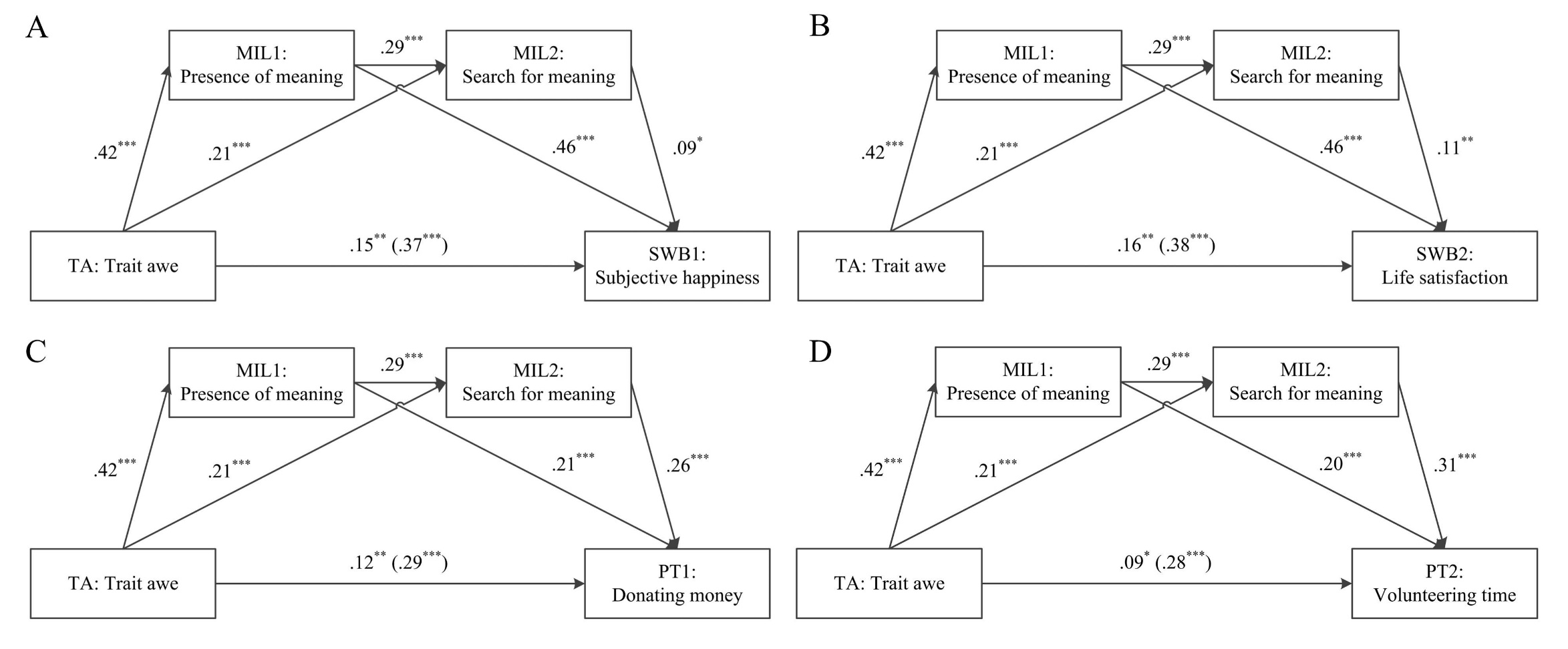


**Figure S3. The Mediation of Meaning in Life between Trait Awe and SWB and Prosocial Tendency.**

**References**

Epskamp, S., Borsboom, D., & Fried, E. I. (2018). Estimating psychological networks and their accuracy: A tutorial paper. *Behavior research methods*, *50*, 195-212. <https://doi.org/10.3758/s13428-017-0862-1>

Epskamp, S., Cramer, A. O., Waldorp, L. J., Schmittmann, V. D., & Borsboom, D. (2012). qgraph: Network visualizations of relationships in psychometric data. *Journal of statistical software*, *48*, 1-18. <https://doi.org/10.18637/jss.v048.i04>

Hayes, A. F. (2013). *Introduction to Mediation, Moderation, and Conditional Process Analysis: A Regression-Based Approach*. The Guilford Press.

Opsahl, T., Agneessens, F., & Skvoretz, J. (2010). Node centrality in weighted networks: Generalizing degree and shortest paths. *Social networks*, *32*(3), 245-251. <https://doi.org/10.1016/j.socnet.2010.03.006>
